# Dissociable effects of age and Parkinson’s disease on instruction-based learning

**DOI:** 10.1093/braincomms/fcab175

**Published:** 2021-08-28

**Authors:** Beth L Parkin, Richard E Daws, Ines Das-Neves, Ines R Violante, Eyal Soreq, A Aldo Faisal, Stefano Sandrone, Nicholas P Lao-Kaim, Antonio Martin-Bastida, Andreas-Antonios Roussakis, Paola Piccini, Adam Hampshire

**Affiliations:** 1 Department of Psychology, School of Social Science, University of Westminster, 115 New Cavendish Street, London, W1W 6UW, UK; 2 The Cognitive, Computational and Clinical Neuroscience Laboratory, Department of Medicine, Imperial College London, London W120NN, UK; 3 School of Psychology, Faculty of Health and Medical Sciences, University of Surrey, Guildford GU2 7XH, UK; 4 Brain and Behaviour Laboratory, Department of Bioengineering, Imperial College London, London W12 0NN, UK; 5 Brain and Behaviour Laboratory, Department of Computing, Imperial College London, London W12 0NN, UK; 6 Behaviour Analytics Lab, Data Science Institute, Imperial College London, London W12 0NN, UK; 7 MRC London Institute of Medical Sciences, London W12 0NN, UK; 8 Neurology Imaging Unit, Division of Neurology, Imperial College London, London W12 0NN, UK; 9 Department of Neurology and Neurosciences, Clinica Universidad de Navarra, Pamplona-Madrid 28027, Spain; 10 UK DRI Care Research & Technology Centre, Imperial College London, London W12 0NN, UK

**Keywords:** Parkinson’s disease, learning, fMRI, MRS, memory

## Abstract

The cognitive deficits associated with Parkinson’s disease vary across individuals and change across time, with implications for prognosis and treatment. Key outstanding challenges are to define the distinct behavioural characteristics of this disorder and develop diagnostic paradigms that can assess these sensitively in individuals. In a previous study, we measured different aspects of attentional control in Parkinson’s disease using an established fMRI switching paradigm. We observed no deficits for the aspects of attention the task was designed to examine; instead those with Parkinson’s disease learnt the operational requirements of the task more slowly. We hypothesized that a subset of people with early-to-mid stage Parkinson’s might be impaired when encoding rules for performing new tasks. Here, we directly test this hypothesis and investigate whether deficits in instruction-based learning represent a characteristic of Parkinson’s Disease. Seventeen participants with Parkinson’s disease (8 male; mean age: 61.2 years), 18 older adults (8 male; mean age: 61.3 years) and 20 younger adults (10 males; mean age: 26.7 years) undertook a simple instruction-based learning paradigm in the MRI scanner. They sorted sequences of coloured shapes according to binary discrimination rules that were updated at two-minute intervals. Unlike common reinforcement learning tasks, the rules were unambiguous, being explicitly presented; consequently, there was no requirement to monitor feedback or estimate contingencies. Despite its simplicity, a third of the Parkinson’s group, but only one older adult, showed marked increases in errors, 4 SD greater than the worst performing young adult. The pattern of errors was consistent, reflecting a tendency to misbind discrimination rules. The misbinding behaviour was coupled with reduced frontal, parietal and anterior caudate activity when rules were being encoded, but not when attention was initially oriented to the instruction slides or when discrimination trials were performed. Concomitantly, Magnetic Resonance Spectroscopy showed reduced gamma-Aminobutyric acid levels within the mid-dorsolateral prefrontal cortices of individuals who made misbinding errors. These results demonstrate, for the first time, that a subset of early-to-mid stage people with Parkinson’s show substantial deficits when binding new task rules in working memory. Given the ubiquity of instruction-based learning, these deficits are likely to impede daily living. They will also confound clinical assessment of other cognitive processes. Future work should determine the value of instruction-based learning as a sensitive early marker of cognitive decline and as a measure of responsiveness to therapy in Parkinson's disease.

## Teaser

Parkin et al. show that a subset of people with Parkinson’s misbind binary discrimination rules in working memory during an instruction-based learning task. These errors were coupled with reduced frontal, parietal and anterior caudate activity when rules were being encoded, as well as reduced GABA levels within the mid-dorsolateral prefrontal cortices.

## Graphical Abstract

**Graphical Abstract:**
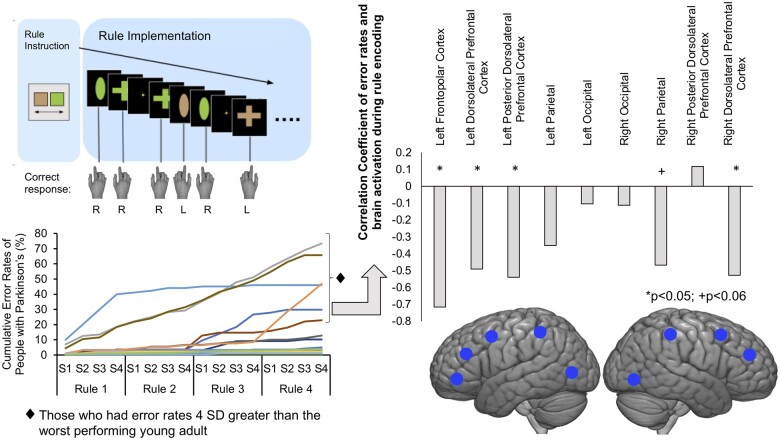

## Introduction

Parkinson’s disease is a multisystem neurological disorder that can disrupt a heterogeneous combination of motor and cognitive functions.^1^^,^^2^ The nature and basis of this heterogeneity is the topic of much debate. Most relevantly, the cognitive domains that are affected in people with Parkinson’s vary in severity, onset and rate of decline.^3^^,^^4^ Deficits in executive, memory or attentional function are common, but do not necessarily co-occur and may relate to degeneration within different brain systems.^5–8^ A substantial proportion of those with Parkinson’s have deficits in these domains at diagnosis, but others remain relatively unaffected through the mid-to-late stage.^9^^,^^10^ Understanding this variability is important as these cognitive deficits impact the patient's ability to lead a full and independent life. Indeed, problems of memory and cognition have been consistently highlighted as a prominent concern for people with Parkinson’s and are predictive of decreased quality of life for both patients and their families/care-givers.^4^^,^^11^ A study polling 1000 patients, carers and healthcare professionals identified mild cognitive problems, such as memory loss, lack of concentration and slowed thinking amongst the top ten unaddressed research priorities for this disorder.^12^

The variability and prominence of cognitive deficits motivate the development of precision diagnostic paradigms that can assess neuropsychological profiles of Parkinson’s at an individual level. Ultimately, this could aid personally tailored treatment approaches, with multi-armed therapies targeting the specific neural systems that are disrupted based on a detailed characterization of impairments. In this study, we examine whether the ability to learn new task rules from explicit instructions provides a sensitive measure of Parkinson’s related cognitive deficits. Tasks that measure aspects of learning in Parkinson’s disease have primarily focussed on reinforcement, the processes that enables goal-directed behaviour to be optimized based on feedback.^13–19^ Performance on these tasks can be impaired in people with Parkinson’s. However, the operational requirements of the tasks are complex. Unlike reinforcement learning, in instruction-based learning (IBL) there is no requirement to explore alternative rules or process feedback contingencies. However, this simplicity belies a complex sequence of psychological and brain processes. Attention must first be orientated to the instruction stimulus, which is parsed and encoded, then applied, initially with support from working memory, prior to consolidation and eventual automatization. This latter phase of consolidation through practice is complex and is associated with a dynamic shift in the involvement of frontoparietal, frontostriatal and default-mode networks in the brain.^20–26^

Given that frontoparietal and frontostriatal systems can be disrupted in Parkinson’s disease,^27–31^ the processes that underlie IBL may be affected. Accordingly, we have reported incidental evidence that people with Parkinson’s are unusually slow to understand the basic operational requirements of an attentional switching task, that is as opposed to showing the expected switching costs.^32^^,^^33^ This slowed task learning was concomitant with reduced frontostriatal activity. Similar to almost all patient assessments, the performance of this task required instructions to be understood, learnt and applied. Thus, a deficit when learning new task rules from explicit instructions are likely to confound patient assessment and have a considerable impact on daily life’ but are yet to be directly explored in this patient group.

To test this hypothesis, a cohort of early-to-mid stage people with Parkinson’s undertook our simple IBL paradigm in the MRI scanner while abstaining from medication. We contrasted their performance and brain activity to that of age-matched controls and younger adults. The task involved learning simple binary discrimination rules, then applying them to sort sequences of object stimuli that differed in colour and shape. First, we sought to confirm that older and younger adults would perform the task with near perfect accuracy,^21^ whilst people with Parkinson’s would have increased error rates. Next, we characterized the nature of observed deficits, by determining whether the errors were consistent, reflecting the misbinding of rules, or variable, indicative of deficits in sustaining these rules in working memory. Then, using fMRI, we examined whether abnormal activity was evident within the frontoparietal or frontostriatal brain areas that are known to be involved in IBL, and determined the stage of the IBL process when abnormalities occur.

Finally, we analysed magnetic resonance spectroscopy (MRS) data from the mid-dorsolateral prefrontal cortex (DLPFC) to determine whether gamma-Aminobutyric acid (GABA) and glutamate, levels differ in individuals who expressed IBL deficits. GABA and glutamate constitute the major inhibitory and excitatory neurotransmitters in the brain and have been associated with working memory function, including working memory capacity^34^^,^^35^ and cognitive decline in older adults.^36^ It has been proposed that these systems underpin cognitive symptoms in neurodegenerative disorders,^37^ and although they have not been the focus of much prior work in Parkinson’s disease, there is evidence that individual differences in levels of GABA predict specific Parkinson’s related symptomology.^38^

## Material and methods

### Participants

Seventeen mild-to-moderate stage participants with Parkinson’s (8 males; mean age: 61 years; range: 47–73) were recruited from a specialist Neurology clinic at Imperial College London NHS trust. Diagnosis was performed by a consultant Neurologist according to the Parkinson’s UK Brain Bank Criteria, excluding other atypical Parkinsonism, history of cognitive impairment, concomitant vascular load and neurodegenerative disorders other than Parkinson’s. Nine participants were receiving dopaminergic therapeutic regimes, they were instructed to withdraw medication 24 h before the assessment for control-release medications or the morning of the study for immediate-release medications. Seven participants were being prescribed combination treatments that included Levodopa, and all nine were receiving drugs that increased dopamine availability in the brain (including dopamine agonists, monoamine oxidase-b-inhibitors, dopa-decarboxylase inhibitors and catechol-o-methyltransferase inhibitors). No adverse side effects to drug withdrawal were reported, in particular, the degree of movement during the MRI assessment did not significantly correlate with Levodopa equivalent dose (LED) in the people with Parkinson’s group, and no exclusions were made due to problematic levels of movement.

Eighteen age-matched older adults (8 males; mean age: 61.3 years; range: 50–73) and 20 younger adults (10 males; mean age: 26.7 years; range: 20–39) were recruited via Imperial College London’s Clinical Research Facility volunteer panel. Exclusion criteria included the presence of psychiatric or neurological disorders, and prescriptions of psychoactive medication. Older adults were screened to ensure they had no family history of Parkinson’s disease. One dataset was excluded from the older adult group because the participant stopped responding and reported falling asleep. The sample size was decided upon based on previous work.^21^

All participants were right-hand dominant with the exception of one older adult. They all had normal or corrected-to-normal vision, spoke fluent English, and had no contraindications that would exclude them from MRI scanning. Written consent was obtained prior to participation. The study was approved by the Imperial College London Ethics Committee according to the Declaration of Helsinki. Participants’ details are summarized in Table 1.

**Table 1:** Demographic and clinical characteristics of people with Parkinson’s and healthy controls

	Young adults	Older adults	People with Parkinson’s
*N*	20	17 (plus 1 excluded)	17
Age (years)	26.7 (5.0)	61.3 (6.8)	61.3 (7.2)
Gender (M:F)	10:10	11:7	8:9
Disease duration (years)			5.0 (3.1)
Age at onset			56.31 (7.65)
Levodopa equivalent daily dosage (LEDD)			475.48 (351.57)
Hoehn and Yahr stage			2.0 (0.42)
UPDRS (off)			40.73 (19.48)
UPDRS III (off)			30.47 (12.10)

Values indicate Mean (SD). There were no significant differences across those with Parkinson’s and older adults on demographic variables. Clinical severity was assessed using the Hoehn and Yahr Scale and the Unified Parkinson's Disease Rating Scale (UPDRS).

### Behavioural task

**Figure 1:**
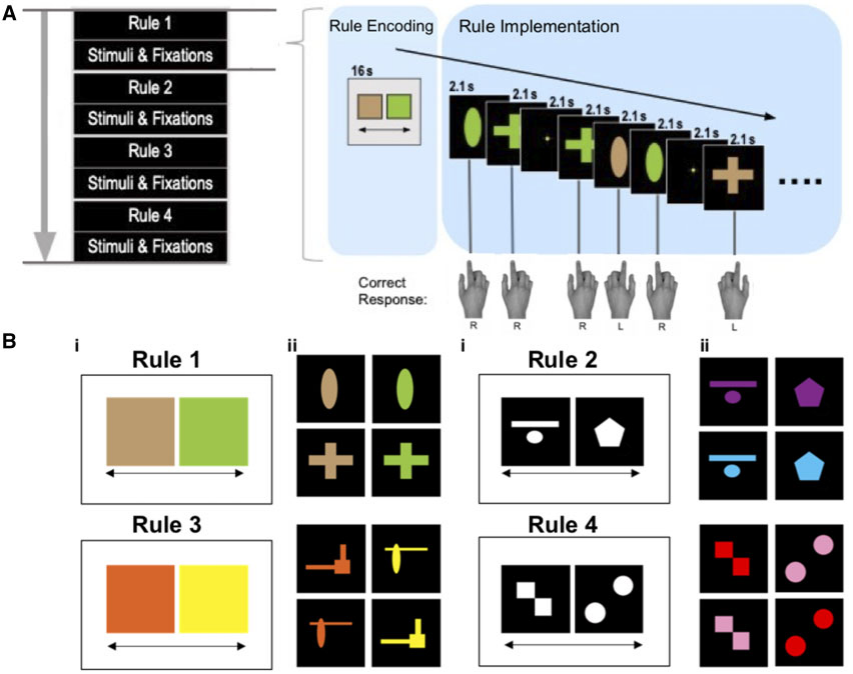
**The Instruction-Based Learning Task.** (**A)** Participants sort coloured shapes according to explicitly instructed discrimination rules. Initially, a binary rule was presented on screen for 16 s (rule encoding phase). Participants then apply this rule to categorize a series of coloured shapes which appear on screen, via a left- or right-hand button press (rule implementation phase). Next, a new rule is shown followed by a novel set of coloured shapes. (**B**) There was a total of four discrimination rules. B (i) presents the pictorial rule instruction slides and B (ii) the corresponding stimulus set.

Participants undertook a paradigm designed to fractionate the component processes of IBL (Fig. 1).^21^ The task was programmed using the Psychophysics Toolbox extension for MATLAB.^39^ Participants were required to categorize stimuli according to binary shape or colour rules. Initially, a pictorial instruction screen displayed the rule as two flankers (e.g. brown = left, green = right), with ‘<-Press left’ & ‘Press right->’ under each object, for 16 s, followed by a 2.1 s fixation cross. Participants then applied this rule to sort a series of centrally presented coloured shapes via a left- or right-hand button press. Button presses were made with the index finger using MRI-compatible response grips (ResponseGrip, NordicNeuroLab, Bergen, Norway). The stimuli varied according to two dimensions, colour (e.g. brown or green) and shape (e.g. cross or circle), with four possible colour–shape combinations per rule (e.g. brown cross, brown circle, green cross and green circle). Stimuli were presented for 2.1 s, in pseudo-randomized sequences of 57 trials per block. A fixation cross, requiring no motor response, was presented instead of a stimulus in 37% of trials. There were four blocks (lasting 2.2 min), each with a unique set of colours and shapes to ensure there was no requirement to override learnt stimulus-rule associations. Before each block visual instructions were presented depicting the current rule for the following block. There were no language elements in the instruction cue. Instead, the relevant stimulus dimension exemplars were provided placed on the corresponding side of the screen. The blocks were arranged in a fixed sequence such that the rules changed across dimensions. No feedback was given during the task. A practice session was completed before entering the scanner, whereby participants read a written description of the task and had the opportunity to ask questions. To ensure that the requirements of the task had been fully understood, participants undertook a demo version with just one block. The experimenter verbally checked that the participant understood what they were being asked to do and how the instruction slides would be presented.

### MRI data

#### Acquisition

Images were acquired using a 3-T Siemens Magnetom Verio MRI scanner with Syngo MR B17 software (Siemens, Erlangen, Germany). T_2_-weighted echo planar images depicting the BOLD contrast were obtained using a 12-channel head coil. Images consisted of 35*3.0 mm slices, with a, 64 × 64 matrix, 192 × 192 × 105 mm field of view, 80° flip angle, 2 s (TR), 30 ms (TE), echo spacing of 0.61 ms, 1906 Hz/Px bandwidth and GRAPPA acceleration factor of 2. A 1 mm structural scan was also collected for each individual, using a MPRAGE T1-weighted sequence which consisted of a, 256 × 240 × 192 matrix, 9° flip angle, 2.25 s (TR), 900 ms (TI), 2.99 ms (TE) and GRAPPA acceleration factor of 2.

The data were pre-processed and analysed in SPM12 (www.fil.ion.ucl.ac.uk/spm) and MATLAB 2016b. Specifically, functional images were slice time and motion corrected and co-registered to the participant’s structural scan. The structural scans from all participants were used to generate a custom template using the DARTEL toolbox.^40^ With this template functional scans were normalized to the standard Montreal Neurological Institute coordinate system, resampled to isotropic 2 mm cubed voxel size, and spatially smoothed with an 8 mm^3^ fullwidth at half maximum Gaussian kernel. Lastly, the data were high-passed filtered to remove low-frequency drifts.

### Statistical analysis

#### Univariate

General linear models were constructed from 12 predictor functions and a constant term at the individual subject level. These consisted of the onset and duration of task events convolved with the canonical haemodynamic response function. Two predictors related to the instruction phase; one captured the entire duration the rule was on screen for (rule encoding: duration 16 s) and the other captured the point at which the rule was first presented (attentional reorienting: modelled as a stick function at onset with a duration of 0). A further four predictors related to rule implementation, when participants made binary discriminations. This was broken down into four consecutive learning stages (∼9 trials each), allowing activation changes to be inferred as behaviour became automatized. Predictors for each stage were modelled as a stick function at the onsets of the individual trials within that stage and with duration = 0. Six nuisance predictors captured *X*, *Y* and *Z* axis translational and rotational head movements.

#### Group analysis

**Figure 2:**
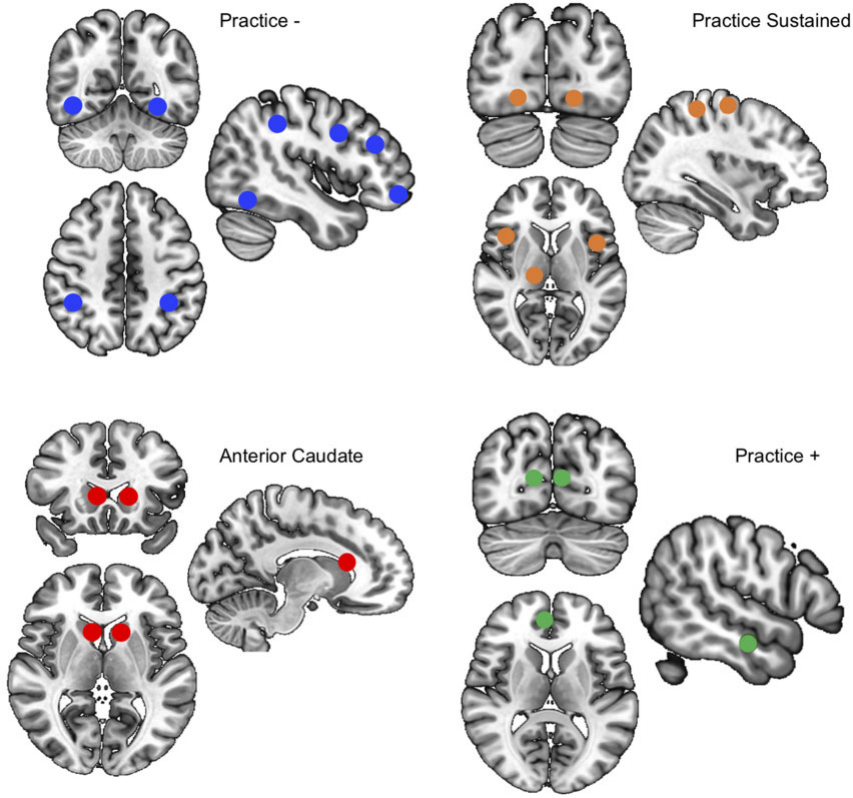
**The location of predefined ROI-sets. There were four ROI-sets based on peak activation coordinates from Hampshire et al.**,^22^ these related to brain regions that were found to disengage with practice (Practice −), increase in activation with practice (Practice +) and those that showed a sustained response throughout rule implementation (Practice Sustained). Bilateral anterior caudate was also included.

Individual level whole brain maps depicting the parameter estimates were exported for group level analysis. Focussed region of interest (ROI) analyses were performed to determine differences in brain activation in Parkinson’s disease in specific regions known to be involved in IBL. ROIs were predefined according to peak activation coordinates from group level contrast maps from Hampshire et al.,^22^ this study had a similar design in that a learning phase was divided into four stages. The contrast maps from Hampshire et al.^22^ were created using the following weighted linear contrasts **(**ROIs are displayed in Fig. 2; coordinates listed in Table 2):

**Table 2:** Pre-defined ROI-sets

Contrast	ROI	Hemisphere	MNI coordinates		
			*x*	*y*	*z*
Practice−	DLPFC	Left	−48	34	22
	DLPFC	Right	42	40	20
	LOFC	Left	−38	48	−8
	LOFC	Right	42	52	−12
	Posterior DLPFC	Left	−44	6	32
	Posterior DLPFC	Right	44	8	38
	PreSMA		0	30	42
	Parietal Cortex	Left	−44	−44	46
	Parietal Cortex	Right	34	−48	48
	LOC	Left	−44	−56	−10
	LOC	Right	36	−46	−16
Practice+	Temporal Cortex	Left	−50	−18	−14
	Temporal Cortex	Right	54	−8	−22
	Occipital Cortex	Left	−10	−72	18
	Occipital Cortex	Right	10	−76	20
	MOFC		−4	48	−10
Sustained	Occipital Cortex	Left	−16	−82	−8
	Occipital Cortex	Right	16	−96	14
	Anterior Insular	Left	−38	14	8
	Anterior Insular	Right	48	12	4
	Inferior Parietal Cortex	Left	−36	−28	44
	Inferior Parietal Cortex	Right	52	−26	44
	Frontal Operculum	Left	−42	−2	10
	Frontal Operculum	Right	42	0	12
	SMA		0	0	48
	Motor Cortex	Left	−38	−20	56
	Motor Cortex	Right	32	−4	54
	Thalamus	Left	−14	−20	6
Caudate	Anterior Caudate	Left	−12	20	4
	Anterior Caudate	Right	12	20	4

These were defined from peak activation coordinates from Hampshire et al.^22^ for the following contrast: Practice −: regions that disengaged with performance. Practice +: regions that increased in activation with practice; Practice Sustained: regions that showed sustained responsiveness throughout rule implementation. Bilateral anterior caudate was also included. DLPFC, dorsolateral prefrontal cortex; L/MOFC, lateral/medial orbitofrontal cortex; LOC, lateral occipital cortex; SMA, supplementary motor area.

**Practice− [contrast across learning stages 3, 1, −1, −3]**: Regions that decreased in activity with practice. ROIs were within the frontoparietal network, specifically the DLPFC, lateral frontopolar cortex, preSMA, parietal cortex and lateral occipital cortex.

**Practice + [contrast across learning stages −3, −1, 1, 3]:** Regions that increased in activity with practice. ROIs were within the default mode network, including the medial orbitofrontal cortex, precuneus and temporal cortex.

**Practice Sustained [contrast across learning stages 1,1,1,1**]: Regions that show sustained activity throughout rule implementation, including the occipital cortex, inferior parietal cortex, anterior insular, frontal operculum and temporal cortex.

An additional ROI within bilateral **anterior caudate** were defined from peak coordinates in the right hemisphere found in Hampshire et al.^22^ In IBL, this region is thought to facilitate the formation of new discrimination rules in working memory^22^ or mediate the implementation of new rules early in the consolidation process.^23^^,^^26^ This region has also been implicated in the cognitive deficits associated with Parkinson’s, in particular hypoactivation in this region has been observed when working out new rules during an Intra-Extra Dimensional Set Shifting task.

Five millimetre radius spherical ROIs were constructed using the MarsBaR toolbox.^23^^,^^26^ The averaged beta weights were calculated across all voxels within each set of ROIs for each subject. These values were analysed at the group level using *t*-tests, correlations and ANOVAs in order to examine the distinct stages of IBL including attention reorienting to instructions, the sustained encoding of novel rules, and across the stages of learning during rule implementation.

#### Voxelwise analysis

In addition, voxelwise group level analyses are reported in supplementary materials. This analysis is applied to confirm that task related patterns of activation were similar to that observed in previous work. Unless otherwise stated, group level statistical maps were generated using a voxelwise thresholding of *P* < 0.01 followed by family wise error cluster correction for the whole brain mass at *P* < 0.05.

### MRS data

#### Acquisition

**Figure 3:**
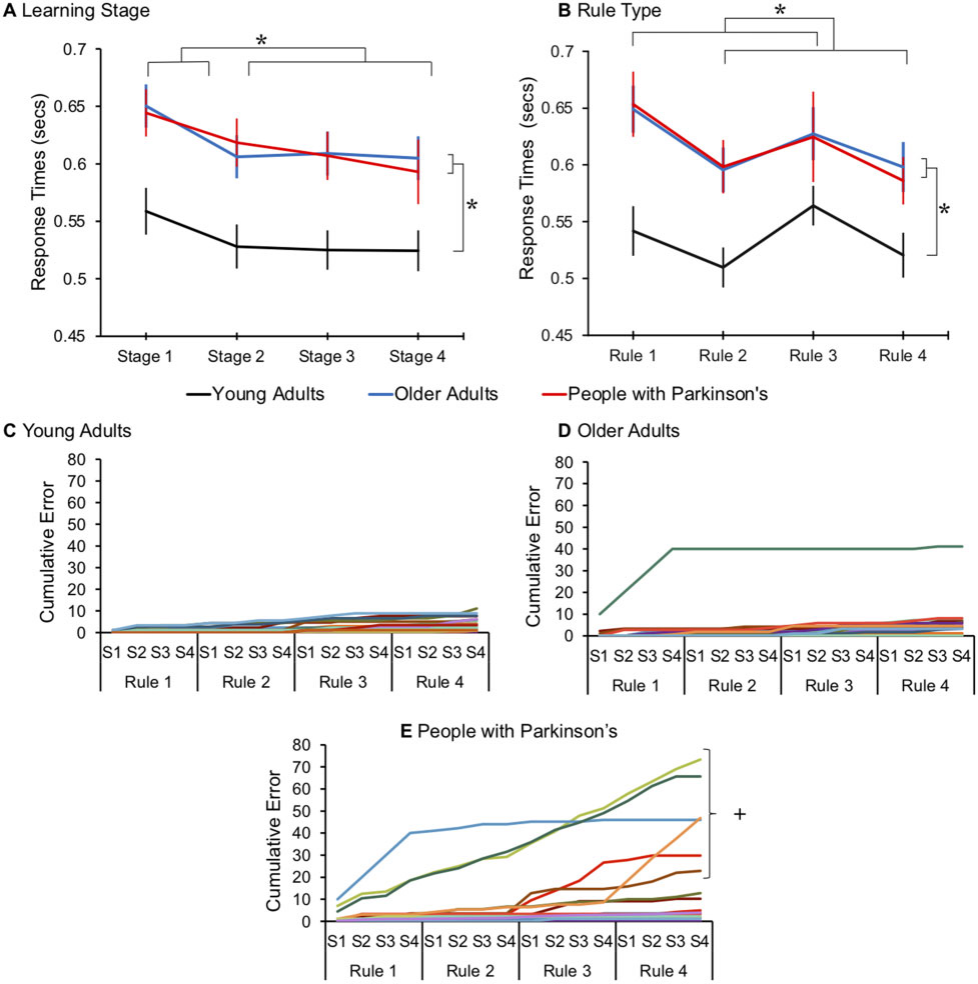
**Behavioural results.** (**A**) Reaction times were significantly faster in young adults, compared to older adults and those with Parkinson’s. All participants were significantly slower to respond during the first quarter of discrimination trials and (**B**) to rules relating to colour compared to shape. (*P < 0.05; two-tailed significance, error bars report the standard error of the mean). (**C**) Single-subject error rates show that all young adults and (**D**) older adults performed the task to a high degree of accuracy (with the exception of one older adult). (**E**) A subset of the Parkinson’s disease group showed increased error rates which reflected a tendency to misbind rules in working memory. (+) Those who had error rates 4 SD greater than the worst performing young adult, these individuals are identifiable at a single-subject level (S1–S4 correspond to sequential learning stages for each block)

MRS data were acquired with a 32-channel head coil, using the MEGA-PRESS method.^41^^,^^42^ A single voxel was positioned in the mid-DLFPC (Fig. 3A). Local shimming was undertaken using the FAST(EST)MAP routine, which performed an automatic first and second order shimming using 2D projections^43^ resulting in water signal linewidths (FWHM) of 8.7–17.9 Hz. Both sequences were part of the Centre for Magnetic Resonance Research Spectroscopy Package, developed by Edward J. Auerbach and Małgorzata Marjańska, provided by the University of Minnesota under a C2P agreement. MEGA-PRESS acquisition parameters included, 20 mm^3^ isotropic voxel, 68 ms (TE), 2 s (TR), 128 averages, 1024 datapoints, 2000 Hz bandwidth, VAPOR water suppression, resulting in an acquisition time of ∼9 min. An additional MEGA-PRESS spectrum without water suppression was also acquired (6 averages). Prior to acquisition, an additional MPRAGE was acquired to guide voxel positioning and quantification of tissue composition. The MPRAGE parameters included, 1 mm^3^ isotropic voxel, 256 × 240 × 160 matrix, 9° flip angle, 2.3 s (TR), 900 ms (TI), 2.98 ms (TE) and GRAPPA acceleration factor of 2. MEGA-PRESS data from three participants with Parkinson’s and one older adult were not acquired due to time constraints.

#### Analysis

MEGA-PRESS spectra were analysed using the Gannet 3.0 software (^44^ RRID: SCR016049). Preprocessing included frequency and phase correction in the time domain using spectral correction, 3 Hz exponential line broadening, and fitting of the choline and creatine signals. This was followed by subtraction of the OFF from ON acquisitions yielding a GABA+ peak at 3 ppm (GABA+ denotes that this signal contains contributions from macromolecules) and Glx (glutamate + glutamine) peak at 3.75 ppm (Fig. 3B). GABA+ and Glx peaks were fitted separately and normalized by the area of Creatine (Cr). Only spectra with relative fit error ≤21% were included. Data from one participant with Parkinson’s and two older adults were excluded based on these criteria. GABA+/Cr or Glx/Cr values of three young adults constituted outliers and were also excluded. Thus, MRS data from 17 young adults, 15 older adults and 13 participants with Parkinson’s was included in the analysis. Gannet routines were employed for voxel registration and tissue segmentation analyses.

### Data availability

Data are available from the authors on reasonable request.

## Results

### Behavioural results

Reaction times and error rates were analysed to determine the relationship between age, Parkinson’s disease and task performance. Trials were sorted into four consecutive learning stages. For each participant, an average was calculated for each stage for each rule. An ANOVA was undertaken with a between-subject factor group (young adults, older adults and Parkinson’s disease), and within-subject factors of rule (1–4) and learning stage (1–4). Bonferroni corrected *post hoc* analyses were performed, unless otherwise stated, and Greenhouse–Geisser correction applied where appropriate.

### Reaction times

There was a significant main effect of group [*F*(2,51) = 6.82, *P* = 0.002], whereby younger adults were significantly faster than older adults (*P* = 0.007) and people with Parkinson’s disease (*P* = 0.009). Older adults and those with Parkinson’s did not differ. There was also a significant main effect of learning stage [*F*(3,153) = 15.41, *P* < 0.001], with participants’ across all groups showing faster responses as the rules were practiced. *Post hoc* tests showed that responses in the first quarter of the task were significantly slower than those made in the later stages (Fig. 3A). There was a significant effect of rule [*F*(1.96,99.9) = 7.02, *P* = 0.002], whereby participants across all groups were slower to respond to colour versus shape rules (Fig. 3B). There were no significant interactions (see Supplementary materials for *post hoc* tests).

### Error rates

Using an ANOVA model of the same design, there was a significant main effect of group [*F*(2,51) = 5.60, *P* = 0.006]; however, unlike response times, those with Parkinson’s made significantly more errors than older (*P* = 0.035) and younger adults (*P* = 0.008). Young and older adults did not significantly differ. There was a significant main effect of learning stage [*F*(2.38,121.66) = 4.40, *P* = 0.010] and a significant interaction of learning stage and group [*F*(4.77,121.66) = 2.55, *P* = 0.033]. When ANOVAs were conducted separately at each learning stage with conditions rule and group, similar main effects of group were evident. In addition, those with Parkinson’s showed a significant effect of learning stage [*F*(3,48) = 3.95, *P* = 0.014], with more errors made during the initial learning stage compared to all other stages (between S1&S2 *P* = 0.041; S1&S3 *P* = 0.024; S1&S4 *P* = 0.021 LSD correction applied), which was not the case for the control groups (Young adults: no significant effect of stage [*F*(3,57) = 2.14, *P* = 0.105]; Older adults: no significant effect of stage *F*(3,48) = 2.051, *P* = 0.119). Error rates did not differ according to the rule type [*F*(1.96,99.92) = 2.43, *P* = 0.095] and there were no other significant interactions of main effects (See Supplementary materials for *post hoc* tests).

**Figure 4:**
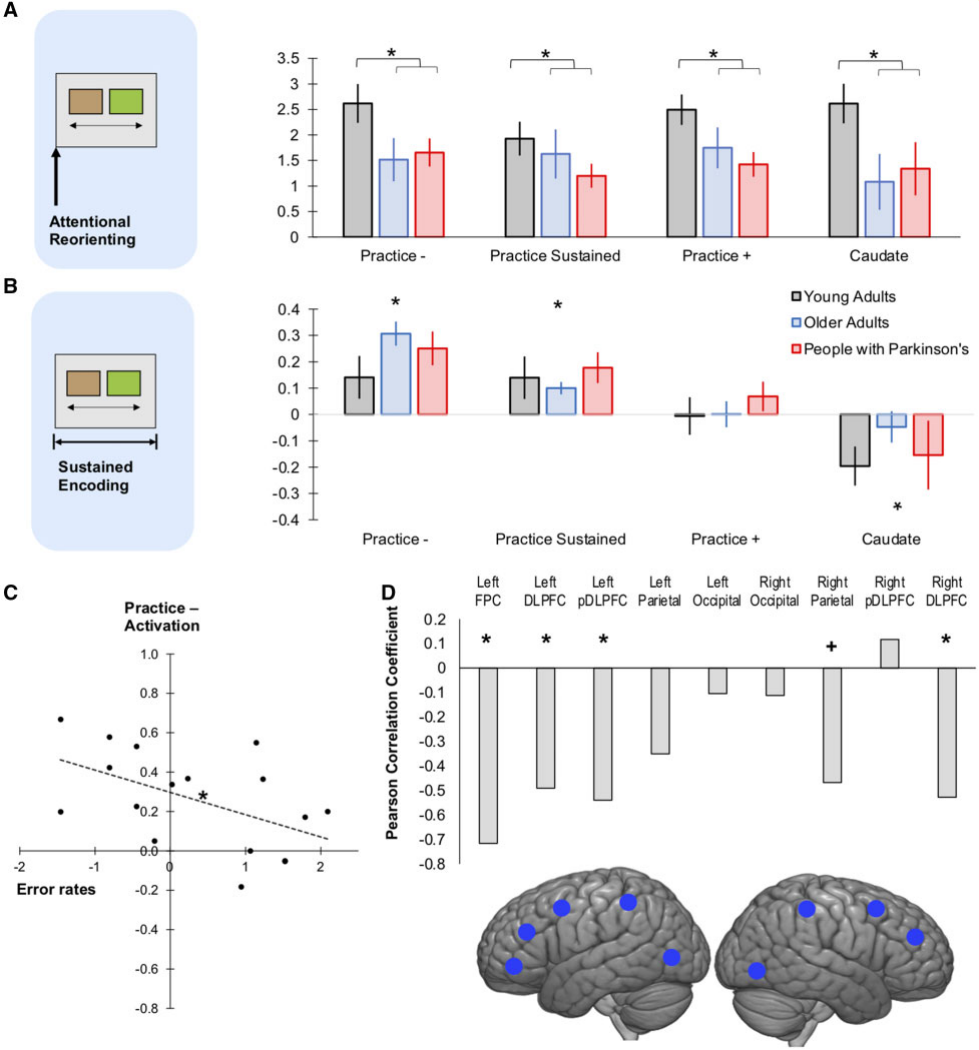
**Mean ROI activation during instruction.** (**A**) Attentional reorienting captured the transient activity at the onset of instruction slide. There was increased activation across all ROI-sets, which was significantly higher in young adults compared to older adults and people with Parkinson’s. (**B**) Sustained encoding captured the entire duration the rule was presented on screen for. Across all participants the Practice− and Sustained ROIs showed increased activation and the anterior caudate showed significant deactivation (*P < 0.05; two-tailed significance, error bars report the standard error of the mean). (**C**) The increased error rate in Parkinson’s disease was associated with reduced activation in the Practice− ROI set (**D**) this related to the ROIs within the frontoparietal cortex (*P < 0.05; +*P* < 0.06).

Overall, all groups performed to a high level of accuracy (mean% accuracy ± SE; young adults: 97.6 ± 2.06, older adults: 96.3 ± 2.24, Parkinson’s disease: 88.0 ± 2.24), indicating that participants understood the general operational requirements of the task. At a single subject level, this was true for every control participant with the exception of one older adult who consistently applied the wrong mapping for the first rule, leading to near 100% error rate for trials of the corresponding block (Fig. 4C/D). This consistent pattern of errors shows they misbound the rule, mapping the features to the wrong responses.

Importantly, the majority of people with Parkinson’s performed near perfectly, with the group level differences driven by a subset who showed much higher error rates, i.e. six participants had errors rates >4 SDs than the worst performing young adult (Fig. 3E). The responses in this subset indicate that they misbound the stimulus-response rules. Two participants inverted the left versus right response mapping, leading to ∼100% errors through the block. Two others sorted the colour-shapes according to the incorrect dimension for one or two rules, leading to chance performance during the subsequent block. The two worst performing individuals operated overall at close to chance. Analysis of their responses showed above chance performance with respect to their consistent application of the same incorrect feature-response mappings (77% *P* < 0.001; 65% *P* = 0.09; probability calculated relative to 1000 random permutations). There were no differences in task performance in those with Parkinson’s disease who abstained from their medication, and no significant correlations of error rates and age or years since diagnosis (see Supplementary materials).

### Neuroimaging results

Brain activation during instruction was examined when (i) initially reorienting attention towards novel rules and (ii) throughout the presentation of the rule slide. ROI analyses were undertaken, whereby, parameter estimates for these contrasts were averaged across all voxels within the four pre-defined ROI-sets which related to regions that showed (i) decreased activation with practice (Practice−); (ii) increased activation with practice (Practice+); (iii) sustained activation with practice (Practice Sustained); and (iv) the anterior caudate. The averaged beta weights were examined in an ANOVA where the within-subject factor was ROI-set, the between-subject factor was group (young adults, older adults and Parkinson’s disease) and average error rates [a rank transformation (arcsine) was applied] were included as a covariate. *Post hoc* comparisons were made with LSD correction.

### Brain activation during the reorienting of attention to novel instructions

#### Younger adults showed significant group differences in brain activation during attentional reorienting

Examination of brain activity when reorienting attention towards a novel instruction slide showed a significant main effect of group [*F*(2,50) = 3.53, *P* = 0.037], no significant main effect of ROI-set [*F*(1.74,87.20) = 1.24, *P* = 0.292] or error rate [*F*(1,50) = 0.58, *P* = 0.449] and no significant interactions of main effects. The main effect of group was driven by younger adults having significantly stronger activation compared to those with Parkinson’s (*P* = 0.020) and older adults (*P* = 0.040), while older adults and those with Parkinson’s did not differ (Fig. 4A). Supplementary analysis of individual ROIs collapsed across group, showed significant activation for all ROIs within each set (with exception of the right anterior caudate) (Supplementary Table 1). Supplementary voxelwise analysis confirmed this increased activation throughout many cortical and subcortical regions at the onset of the instruction slide (Supplementary Fig. 1A and Supplementary Table 3). Thus, there was a strong widespread spike in activity during attentional reorienting to new instructions, which was sensitive to age but not to Parkinsonism.

### Brain activation during rule encoding

When brain activation during the sustained encoding of instructions was compared across groups, there was a significant main effect of ROI-set [*F*(2.51,125.39) = 36.30, *P* < 0.001], no significant main effect of error rate [*F*(1,50) = 2.69, *P* = 0.107], and critically, a significant interaction of ROI-set and error rate [*F*(2.51,125.39) = 3.91, *P* = 0.015]. There was no significant effect of group [*F*(2,50) = 0.64; *P* = 0.531], and no other significant interactions of main effects.

#### Significant correlations of error rate and encoding related activation in Parkinson’s disease

The interaction of error rates and ROI-set related to the Practice− ROIs. When focussing on those with Parkinson’s, there was a significant negative correlation of error rate and activation within the Practice− ROI-set [*r*_(17)_ = −0.52, *P* = 0.031]. Additional analysis showed that this related most robustly to ROIs within the frontoparietal cortex (Fig. 4C and D). There was a subthreshold trend in the same direction for activation within the anterior caudate ROIs [*r*_(17)_ = −0.46, *P* = 0.065]. There are no other significant correlations between error rates and brain activity during encoding across the ROI-sets for healthy controls (see Supplementary materials). Activation within the Practice Sustained [*r*_(17)_ = 0.13, *P* = 0.627] and Practice+ [*r*_(17)_ = −0.19, *P* = 0.466] ROI-sets did not correlate with error rates. Therefore, the high rate of misbinding errors observed in Parkinson’s disease related to reduced activation within the frontoparietal cortices when encoding novel rules.

#### Differential sensitivities of the ROIs during rule encoding

Overall, there was a differential response per ROI-set during rule encoding (Fig. 4B). The Practice– [*t*_(53)_ = 5.75, *P* < 0.001] and Practice Sustained ROIs [*t*_(53)_ = 4.00, *P* < 0.001] showed heightened activity during rule encoding. For the Practice− ROIs, this increased activation was evident for all ROIs with the exception of those within the right prefrontal cortex. For Practice Sustained ROIs, the significant activation was focussed within bilateral occipital cortex and right motor cortex (Supplementary Table 1). There was no significant activation in the Practice+ ROI-set [*t*_(53)_ = 0.50, *P* = 0.620], and the anterior caudate ROIs showed significant deactivation [*t*_(53)_ = −2.68, *P* = 0.010] during rule encoding. See Supplementary materials for voxelwise analysis of the regions active and deactive during rule encoding (Supplementary Fig. 1B and C; Supplementary Table 4).

### Brain activation during rule implementation

#### There were no significant group differences in brain activation across the learning curve

Responses during rule implementation were examined to determine whether there were differences in learning-related brain activity in Parkinson’s disease. Parameter estimates were averaged for each of the four learning stages. The ANOVA model was similar to that described previously with the addition of the within-subject factor learning stage (1–4). Results showed there was no significant main effect of learning stage [*F*(2.22,110.85) = 1.17; *P* = 0.318], a significant effect of ROI-set [*F*(2.22,111.25) = 57.42; *P* < 0.001] and a significant interaction of learning stage with ROI-set [*F*(4.47,223.23) = 17.90; *P* < 0.001]. Importantly, neither the main effect of group [*F*(2,50) = 0.02; *P* = 0.985] nor error rates [*F*(1,50) = 1.75, *P* = 0.192] were significant and there were no significant interactions of these factors. This further accords with the view that errors in those with Parkinson’s related to abnormal brain activity for the encoding as opposed to application stage.

#### Differential sensitivities of ROIs across the learning curve

**Figure 5:**
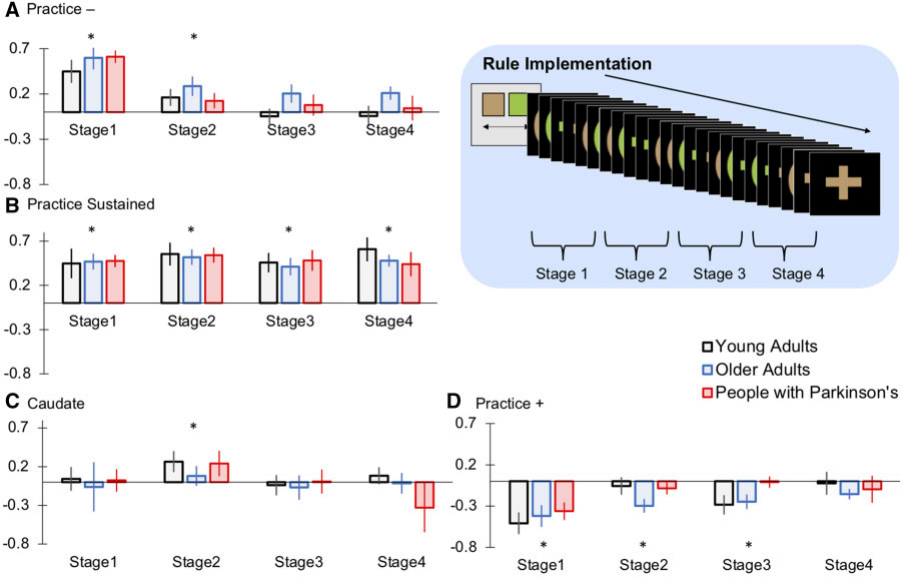
**Mean ROI activation across the learning curve**. **(A)** Practice− ROIs showed initial activation which decreased as rules became familiar. **(B)** The Practice Sustained ROI-set showed increased activation throughout all learning stages. **(C)** The anterior caudate ROI showed increased activation during the second quarter of rule implementation trials. **(D)** The Practice + ROIs showed initial deactivation which increased to baseline as rules became familiar. This pattern of activation did not differ across the young adults, older adults or people with Parkinson’s (*P < 0.05; two-tailed significance. Error bars report the standard error of the mean).

We observed a similar learning-related dissociation of the frontoparietal and default mode network to that reported previously.^21^^,^^22^ ANOVAs conducted separately for each ROI-set showed significant differences across the learning stages for the Practice− [*F*(2.31, 122.39) = 25.21, *P* < 0.001) and Practice+ ROIs [*F*(2.49, 131.85) = 8.49, *P* < 0.001). The Practice− ROIs initially showed increased activation and returned to the resting baseline with practice. The Practice+ ROIs showed initial significant deactivation and progressed back to baseline with practice. The Practice Sustained ROIs showed significant activation throughout all four task implementation stages with no significant differences across the learning stages [*F*(2.53,134.03) = 1.28, *P* = 0.284]. The caudate ROIs showed significant activation at stage two only (Fig. 5) (see Supplementary material for post-hoc tests and Supplementary Table 2 for analysis of individual ROIs within each set). These results highlight that the process of applying and consolidating discrimination rules during IBL involves a shifting balance of brain regions, and that this is largely retained in older adults and people with Parkinson’s. See Supplementary materials for voxelwise analysis of brain regions that showed increased, decreased and sustained activation during rule implementation (Supplementary Fig. 2 and Table 5–7).

### GABA and glutamate analysis

**Figure 6:**
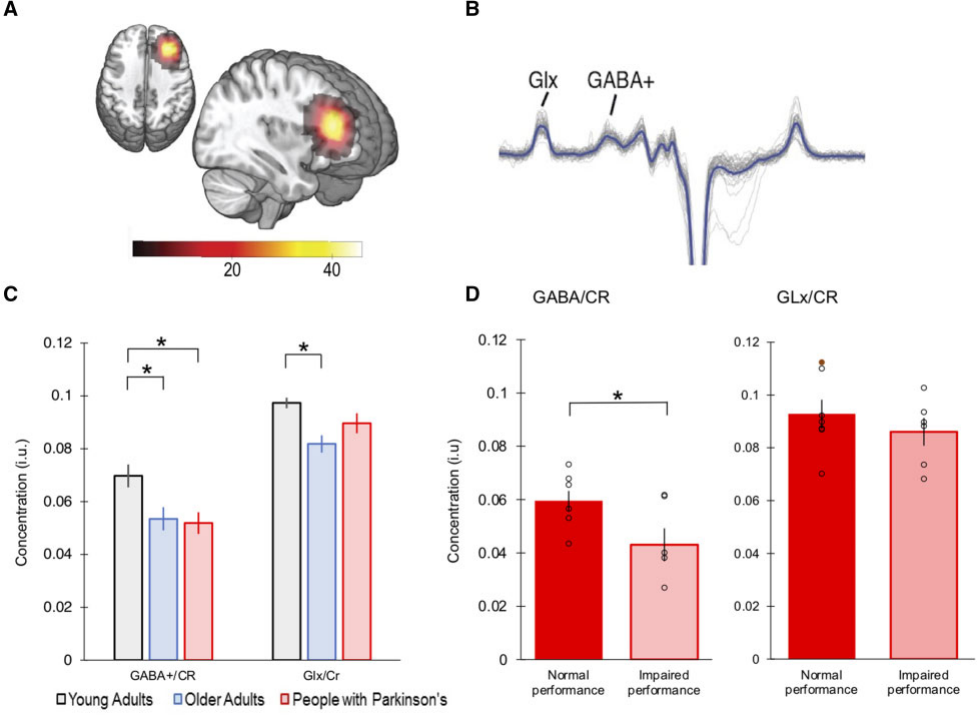
**MRS data.** (**A**) Voxel position across all subjects (the colour bar indicates the number of subjects). (**B**) GABA-edited MEGA-PRESS spectra from the DLPFC. The spectral data are plotted in grey and the average spectrum across all participants is overlaid in blue (for visualization purposes only). (**C**) Measurements of GABA+/Cr and Glx/Cr across groups. (**D**) Measurements of GABA+/Cr and Glx/Cr for those with Parkinson’s who had normal and impaired IBL task performance. Plots represent mean concentrations (*P < 0.05; two-tailed significance. Error bars report the standard error of the mean).

We examined, on a preliminary level, whether GABA+/Cr or Glx/Cr levels differed between groups and whether they were associated with the observed IBL deficits in Parkinson’s disease. As measurements of metabolites are sensitive to relative tissue fractions of CSF, grey matter and white matter within the measured voxel, we examined whether anatomical differences within the DLPFC voxel confounded the measurements of GABA+ and Glutamate. ANOVAs for grey and white matter indicate that these fractions did not significantly differ between groups [Grey matter: *F*(2,42) = 0.108, *P* = 0.898; White matter: *F*(2,42) = 1.05, *P* = 0.358]. There was a group difference for CSF [*F*(2,42) = 5.76, *P* = 0.006], whereby people with Parkinson’s and older adults had significantly increased CSF compared to younger adults. Next, ANCOVAs using CSF as a covariate were undertaken to investigate whether GABA+/Cr or Glx/Cr differed across groups (Fig. 6C). There was a main effect of group on GABA+/Cr [*F*(2,42) = 5.46, *P* = 0.008], with reduced GABA+/Cr levels in older adults and people with Parkinson’s when compared to younger adults. The analysis for Glx/Cr showed a main effect of group [*F*(2,42) = 7.41, *P* = 0.002], with reduced Glx/Cr levels only in older adults compared to younger adults (see Supplementary materials for *post hoc* tests).

To investigate whether the deficits in IBL in Parkinson’s disease were associated with neurotransmitter changes in the DLPFC, we performed a multiple linear regression analysis. The predictors included group (Parkinson’s disease versus older adults), CSF, GABA+/Cr and Glx/Cr concentration ratios. There was only a significant relationship between impairment and GABA+/Cr (*P* = 0.031; all other predictors *P* > 0.08). The adjusted *R*^2^ value was 0.268. *Post hoc* tests showed that GABA+/Cr concentrations within the DLPFC were significantly lower in people with Parkinson’s who tended to misbind the discrimination rules [*t*_(11)_ = −2.36, *P* = 0.040] (Fig. 6D).

## Discussion

This is the first study to directly investigate instruction-based learning deficits in Parkinson’s disease. The results support our hypothesis that the ability to learn new task rules from explicit instructions is substantially disrupted, with approximately one-third of our early-to-mid stage Parkinson’s disease group exhibiting an abnormally high error rate. This may reflect a distinct behavioural subtype of people with Parkinson’s who are prone to misbinding task rules in working memory.

The fact that IBL is operationally simple yet involves a sequence of neural processes that can be clearly delineated across time^20–26^ holds untapped potential to further elucidate the nature of impairments in clinical populations. Here, the behavioural and brain imaging results converge in support of a misbinding mechanism. Specifically, the pattern of errors seen in some people with Parkinson’s was highly consistent and primarily focussed on a subset of the four rule blocks. Therefore, even when the encoded rules were erroneous, participants were able to maintain and apply them consistently throughout the block as per the operational requirements of the task. Furthermore, in the people with Parkinson’s showing high erroneous responses reduced frontoparietal functional activation was evident selectively throughout the encoding phase, but not during the discrimination trials or when the instruction slide was first presented. Again, this accords with abnormal processing of the instructions.

The focus on the encoding phase warrants further discussion. One might have expected abnormal activity to be evident in Parkinson disease when rules are being applied. There is a delicate and shifting balance in the involvement of frontoparietal and striatal brain regions as the discrimination rules become established through a process of repetition.^20–26^ In this respect, it is interesting that previous studies investigating the precision of working memory representations (i.e. delayed reproduction paradigms) have shown an increase in forgetting errors in those with Parkinson’s. This has been attributed to a failure to maintain as opposed to bind working memory representations.^45^^,^^46^ It is important to note that in our paradigm, working memory maintenance load and the necessary precision of working memory representation was minimal by design. There were only two mappings and the stimuli were highly discriminable. Furthermore, the rules were consolidated through practice as opposed to maintained over a lengthy delay. Therefore, deficits observed here, which related to the formation of visual and motor representations in working memory, may be quite distinct from those relating to working memory capacity or fidelity.

Our results accord well with previous observations that people with Parkinson’s, unlike controls, are still learning the overarching operational requirements of a reinforcement learning paradigm even after they have been instructed^32^^,^^33^ and have problems using prior information to guide decision-making.^16^ More broadly, abnormal frontostriatal and frontoparietal activity has been previously implicated in Parkinson’s disease with impairments linked to deficits in working memory, planning, response inhibition,^3^ executive functioning^7^ and general cognitive decline.^47^ It remains unclear the extent to which these tasks measure the same underlying frontostriatal disruption, or whether they capture distinct behavioural profiles of Parkinson’s that have bases in different neural circuits. It is notable though that the performance of all these tasks is contingent on the ability to learn the operational requirements from explicit instructions. Consequently, it seems sensible to suggest inclusion of an IBL paradigm within broader computerized assessment batteries, to determine whether the ability to understand instructions is a barrier to interpreting the results of other cognitive tests.

Indeed, in support of the practical utility of simple IBL, the error rates for individuals who showed an IBL deficit was 4 SD higher than the worst performing young adult. Conversely, the majority of those with Parkinson’s achieved ∼100% accuracy. Tasks that provide a binary classification of Parkinson’s disease according to whether a deficit is evident are rare. Given that the deficits relate to the encoding as opposed to the application phase, it should be possible to develop a short form of the IBL paradigm that has more rules, but fewer trials, saving time and increasing measurement accuracy.

On a mechanistic level, the results align well with studies that have implicated frontoparietal networks in cognitive flexibility.^23^^,^^24^^,^^48–51^ For example, Muhle-Karbe et al.^52^ showed that newly encoded task instructions could be decoded from fMRI activity patterns within the frontoparietal cortex, highlighting a role in the preparatory coding of new behaviours. In addition, it has been proposed that the anterior caudate supports the gating of task relevant information when forming mental representations^53^ and some forms of task switching.^54–56^ This is compatible with the observed association between misbound rules and a trend towards abnormal frontostriatal activity.

The MRS results provide intriguing preliminary evidence that abnormal GABA levels may have a role in the observed IBL deficits. Specifically, the misbinding errors were associated with reductions in GABA, but not Glutamate, in the mid-DLPFC. To date, there has been very little *in-vivo* research into GABA and glutamate systems in Parkinson’s. However, there is a convergence of evidence with the post-mortem research, where alterations in ex-vivo DLPFC GABA levels^57^ have been reported. More broadly, previous research has shown DLPFC GABA concentrations predict working memory capacity^34^^,^^35^ and cognitive decline in older adults.^36^ Furthermore, abnormal GABA has been hypothesized to underpin cognitive symptoms in neurodegenerative disorders.^37^ Altered levels of GABA in midbrain structures and the basal ganglia have been reported in people with Parkinson's.^58^^,^^59^ Perhaps most intriguingly, one of the few cortical MRS studies in Parkinson’s disease, focussed on the occipital lobes,^38^ reported that although GABA levels were unchanged overall individual variability was predictive of hallucination symptoms.

A number of mechanisms could conceivably underpin the relationship observed between reduced DLPFC GABA and misbinding errors. The reductions in GABA may reflect degraded inputs into the working memory network that DLPFC is part of. This would be comparable to the interpretation proposed by Firbank et al.^38^ for their occipital cortex results, and with work reporting reductions in occipital GABA following eye occlusion.^60^ Alternatively, a reduction in GABA might disrupt the excitatory/inhibitory balance of activity within the DLPFC, which has been proposed to influence the selectivity of working memory representations.^61^ Also, reduced GABA may disrupt the capacity of the DLPFC to synchronize with other brain regions. Further work is needed to replicate our findings and to characterize the underlying mechanism.

While we used a well-established approach, there are some limitations to the MRS methodology. Firstly, our observations come from the measurements of one ROI; consequently, we do not know whether the observations would extend to other areas important for rule encoding in IBL. Secondly, our signal is a combination of GABA and macromolecules that allowed us to maximize the signal-to-noise ratio; however, macromolecules have been shown to increase with ageing^62^ and thus changes in GABA might be underestimated in this study.

One of the reviewers noted that pre-cluster corrected voxelwise analysis threshold of 0.01 is below that recommended in one article.^63^ Here, the purpose of this analysis was to replicate the previously reported pattern of activity per task condition,^21^ therefore, we retain the same correction criteria. We note though that increasing voxelwise threshold to *P* < 0.001 does not change the main activation peaks or inference.

Finally, we noted the double dissociation between the effects of age and Parkinson’s on response time and accuracy during IBL, which extend with broader dissociations in the literature.^64^^,^^65^ Both those with Parkinson’s and older adults were slower than young adults. Concomitantly, there was a general effect of age on the transient activation that occurred with the onset of the new instruction slide, with attenuated responses in older adults and those with Parkinson’s. This spike of activity accords with our previous work,^21^ it may reflect the reorientation of attention to the instruction slide^66^^,^^67^ and that ageing impacts both the intra and inter connections of cortical networks that support higher order cognition.^68^ The dissociation stands counter to the notion that cognitive deficits in Parkinson’s disease can simply be considered an accelerated form of normal ageing.^69^

In summary, our results support that a substantial proportion of early-to-mid stage people with Parkinson’s have deficits in IBL, with implications for patient assessment and daily function. This deficit is concomitant with abnormal activity and reduced GABA levels within areas of the brain that are associated with working memory. It appears to reflect a tendency to misbind explicitly instructed stimulus-response rules. Future work should extend these findings in a larger population to determine the relationship between IBL and other cognitive deficits, and to examine more closely the relationship of IBL deficits to the disruption of cortical networks and neuromodulatory systems. Longitudinal work should determine the value of IBL when identifying clinically at-risk patients and monitoring the progression and response to therapy of cognitive deficits. Finally, given the reliance of most assessment paradigms on instructions, we recommend including a brief IBL test when assessing behavioural deficits in Parkinson’s disease, to ensure that the results are not confounded.

## Supplementary material

Supplementary material is available at *Brain Communications* online.

## Supplementary Material

fcab175_Supplementary_Data

## Abbreviations

ANCOVA =: analysis of covariance
Cr =: Creatine
DLPFC =: dorsolateral prefrontal cortex
GABAx =: gamma-Aminobutyric Acid (+ macromolecules)
Glx =: Glutamate + GlutamineF
IBL =: instruction-based learning
LED =: Levodopa equivalent dose
LSD =: least significant difference
MPRAGE =: Magnetization Prepared—RApid Gradient Echo
MRS =: magnetic resonance spectroscopy
ppm =: parts per million
ROI =: region of interest
SD =: standard deviation
SMA =: supplementary motor area
