# Supplementary material for: Dissociable effects of age and Parkinson’s disease on instruction-based learning
Source: Brain Commun. 2021 Aug 28;3(3):fcab175. doi: 10.1093/braincomms/fcab175 (PMC8410985; doi:10.1093/braincomms/fcab175)
Supplement: fcab175_Supplementary_Data [file fcab175_supplementary_data.docx]

**Supplementary Materials**

**Behavioural Analysis**

**Reactions times**

Post hoc tests of reaction times showed that discriminations of objects presented in the first quarter of the task (stage 1) were significantly slower than those made in the later stages (i.e., between stage 1&2 (p<0.001); stage 1&3 (p<0.001); stage 1&4 (p<0.001)). In addition, participants across all groups were slower to respond to the rules relating to colour (rule 1 & 3) compared with those relating to shape (rule 2 & 4) (i.e., there were significant differences between rule 1&2 (p=0.005); rule 1&4 (p=0.009); as well as rule 3&2 (p=0.002); and rule 3&4 (p=0.022)).

**Error Rates**

ANOVAs were conducted separately at each learning stage with conditions of rule and group, similar main effects of group were evident at all stages (stage1: F_(2,51)_=5.94 p=0.005; stage2: F_(2,51)_=5.50 p=0.007; stage3: F_(2,51)_=4.46 p=0.016; stage4: F_(2,51)_=5.07 p=0.010). ANOVAs were also conducted separately for each group with conditions of rule and stage, people with Parkinson’s showed a significant effect of learning stage (F_(3,48)_=3.95 p=0.014) whereby significantly more errors were made during the first quarter of the task compared to all other stages (between S1&S2 p=0.041; S1&S3 p=0.024; S1&S4 p=0.021 LSD correction applied). There was no significant effect of learning stage or interactions in the healthy control groups.

There were no differences in error rates (t_(15)_=0.89 p=0.388) or reaction times (t_(15)_=-0.45 p=0.658) in those with Parkinson’s who abstained from their medication vs those who were unmedicated. There were no significant correlation between error rates and age (r_(17)_= -0.09 p=0.718) or years since diagnosis (r_(17)_= -0.08 p=0.769). There were no significant correlation between reaction time and age (r_(17)_=-0.28 p=0.278) or years since diagnosis (r_(17)_=-0.28 p=0.281).

**Neuroimaging Results**

**ROI analysis during rule encoding**

In people with Parkinsons ~~t~~here was a significant negative correlation of error rates and activation during rule encoding within the left frontopolar cortex (r_(17)_=-0.72 p=0.001), bilateral mid-DLPFC (left: r_(17)_=-0.49 p=0.045; right: r_(17)_=-0.53 p=0.029), left posterior DLPFC (r_(17)_=-0.54 p=0.025) and a subthreshold trend for the right parietal cortex (r_(17)_=-0.47 p=0.058). Activation in the lateral occipital cortices (left: r_(17)_=-0.10 p=0.691; right: r_(17)_=-0.11 p=0.670), the right posterior DLPFC (r_(17)_=0.12 p=0.656) and left parietal cortex (r_(17)_=-0.35 p=0.167) did not correlate with error rates. There are no other significant correlations between error rates and brain activity during encoding across the ROI-sets for healthy young controls (Practice - : r_(20)_ 0.105 p= 0.661: Practice Sustained r_(20)_= -.092 p=0.701 Practice +: r_(20)_= 0.058 p=0.808 Caudate r_(20)_=-0.94 p=0.693) and for older adults’ controls (Practice - : r_(17)_=-0.117 p=0.656 : Practice Sustained r_(17)_ =-0.332 p=0.193 Practice +: r_(17)_=-0.413 p=0.099 Caudate r_(17)_ =-0.407 p=0.105).

**ROI analysis during rule implementation**

One sample t-tests were used for post hoc analysis. The Practice- ROIs showed increased activation at learning stage 1 and stage 2 (stage 1: t_(53)_=8.43 p<0.001; stage 2: t_(53)_=3.54 p=0.001), which progressed back to baseline with practice (during stage 3: t_(53)_=1.30 p=0.199 and stage 4: t_(53)_=0.96 p=0.340). The Practice+ ROI-set showed initial significant deactivation (stage 1: t_(53)_= -6.23; p<0.001; stage 2: t_(53)_= -2.73; p=0.009; stage 3: t_(53)_= -3.17; p=0.003) which progressed back to baseline with practice (stage 4 t_(53)_=-1.19 p=0.238). The Practice Sustained ROIs showed significant activation throughout all four learning stages (stage 1: t_(53)_= 6.60 p<0.001; stage 2: t_(53)_=9.30 p<0.001; stage 3: t_(53)_=7.61 p<0.001; stage 4: t_(53)_= 7.68 p<0.001). The Caudate ROI showed significant activation at learning stage 2 only (t_(53)_=2.40 p=0.020).

**Voxelwise Analysis**

**Brain activation during the reorienting of attention to novel instructions:** Voxelwise analysis showed heightened activation throughout many cortical and subcortical regions at the onset of the instruction slide ***(*Supplementary Table 5*;* Supplementary Fig.1A)**. This related to areas within the ROI sets, including large swathes of the frontoparietal cortex and the default mode network including the medial temporal lobes, precuneus, medial prefrontal cortex and inferior parietal lobules. The caudate was also activated bilaterally, as was the premotor and occipital cortices. In line with the ROI results, there were no significant voxels when comparing people with Parkinson’s to older adults during attentional reorienting.

**Brain activation during rule encoding:**

Regions that showed increased activation during rule encoding included large swathes of the left prefrontal cortex, with a smaller cluster of activation in the right middle frontal gyrus. There were also significant activation in the supplementary motor areas, the occipital cortices and thalamus **(Supplementary Fig.1B; Supplementary Table 6A)**. In contrast, regions of the default mode network within the medial frontopolar cortex showed sustained deactivation, as well as regions within the middle and superior temporal cortex and corpus callosum **(Supplementary Fig.1C, Supplementary Table 6B).**

**Brain activation during rule implementation**

**Regions that decreased in activity with practice:**

Whole brain analysis confirmed that the regions that disengaged with practice (contrast across learning stages: 3,1,-1,-3; **Supplementary** **Table 5; Supplementary Fig.2B**) were within the frontoparietal cortices, including inferior and middle frontal gyrus, inferior parietal cortices, as well as supplementary motor and motor areas and inferior occipital cortices. The insula cortex also showed reduced engagement as rule implementation became automatic. For completeness, we examined the individual ROIs within the predefined sets that were active for this contrast (**Supplementary Table 2)**, and confirmed all ROIs within the Practice- set to show significant activation which was not the case for any ROIs in other sets**.**

**Regions that increased in activity with practice:**

Brain regions that showed the opposite pattern of increasing activation with practice (contrast across learning stage: -3,-1,1,3; **Supplementary Table 6; Supplementary Fig.2A)** confirmed extensive activation within the medial occipital cortices (extending to the calcarine, lingual and cuneus), and also highlighted significant clusters within the medial orbitofrontal cortices, more ventral to the predefined ROI. When examining the responses of individual ROIs within the sets activated for this contrast, the significant engagement of the Practice+ set was driven by activation within the occipital cortex ROIs (**Supplementary** **Table 2).**

**Regions that showed sustained activation throughout task performance:**

Lastly regions that showed sustained activation during rule implementation (contrast across learning stages: 1,1,1,1) (**Supplementary** **Table 7; Supplementary Fig.2C)** were centered within the occipital and parietal areas. Areas within the bilateral thalamus and insula were also active. The middle frontal gyrus showed a sustained response which did not survive FWE correction. When examining the individual ROIs within each set active for this contrast, all ROIs within the Practice Sustained ROI set were active, as well as the bilateral occipital, parietal and left posterior DLPFC ROIs from the Practice- set. None of the more anterior frontal ROIs showed significant activation (**Supplementary** **Table 2).**

**GABA and Glutamate analysis**

Participants with Parkinson’s and older adults had significantly increased CSF compared to younger adults, while those with Parkinson’s and older adults did not differ (older vs younger: p=0.018; Parkinson’s disease vs younger: p=0.015; older vs Parkinson’s disease: p=0.983; p-values adjusted using Tukey HSD). Post hoc t-tests also revealed reduced GABA+/Cr in older adults and people with Parkinson’s when compared to younger adults (older vs younger: p=0.024; Parkinson’s disease vs younger: p=0.016; older vs Parkinson’s disease: p=0.964; p-values adjusted using Tukey HSD). The analysis for Glx/Cr showed reduced levels in older adults compared to younger adults (older vs young: p=0.001; Parkinson’s disease vs young: p=0.180; older vs Parkinson’s disease: p=0.186; p-values adjusted using Tukey HSD).

**Supplementary Figure 1: Voxelwise whole brain analysis confirming expected pattern of activation during instruction, including participants from all groups (n=54) A)** Shows brain regions active during initial attentional reorienting, and B**)** sustained activation and C) deactivation during rule encoding. All are rendered with p <0.05 FWE cluster correction after initial voxelwise thresholding at p <0.01.


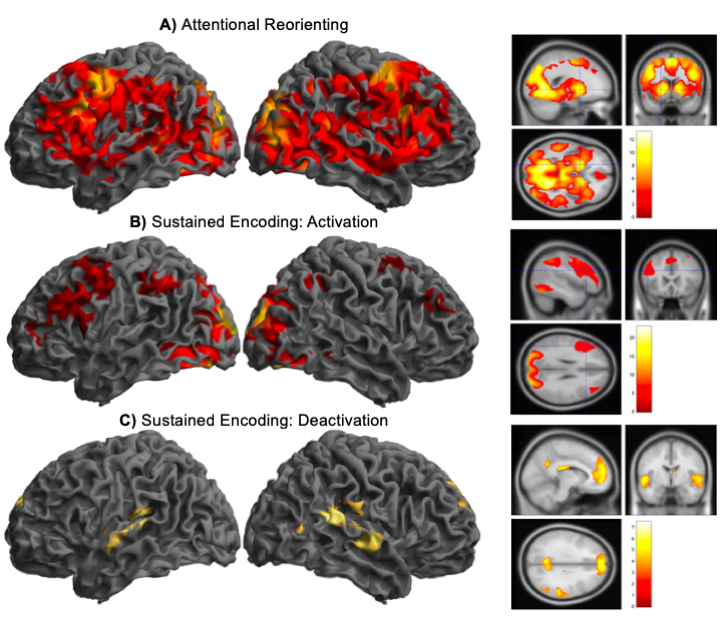


**Supplementary Figure 2:** **Voxelwise whole brain analysis confirming the expected pattern of effects of practice on activation during rule implementation, including participants from all groups (n=54)**. **A)** **Practice +** shows brain regions which increase in activation with practice.  **B) Practice -** shows regions which decrease in activation with practice. **C)** **Practice Sustained** show regions that show sustained activation during performance of the task. All are rendered with p <0.05 FWE cluster correction after initial voxelwise thresholding at p <0.01.


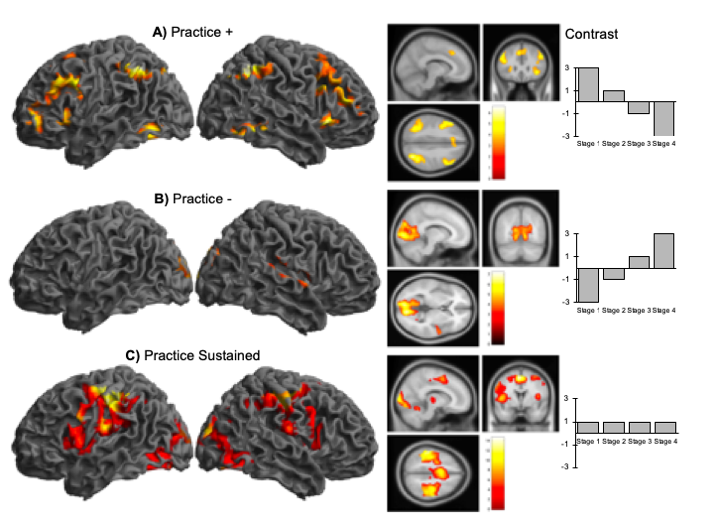


**Supplementary Table 1:** Supplementary analysis of individual ROIs within each set for brain activation during attentional reorienting and the sustained encoding of instructions. *denotes significance at p<0.05.

|  | **ROI** | **Attentional Reorienting** | | **Sustained**  **Encoding** | |
| --- | --- | --- | --- | --- | --- |
|  |  | **t** | p | **t** | **p** |
| Practice - | Left LOC | 5.84 | **<0.001*** | 6.61 | **<0.001*** |
|  | Right LOC | 9.44 | **<0.001*** | 4.57 | **<0.001*** |
|  | Left Parietal | 7.25 | **<0.001*** | 7.95 | **<0.001*** |
|  | Right Parietal | 7.01 | **<0.001*** | 5.83 | **<0.001*** |
|  | Left DLPFC | 6.47 | **<0.001*** | 5.25 | **<0.001*** |
|  | Right DLPFC | 5.70 | **<0.001*** | 2.48 | 0.181 |
|  | Left pDLPFC | 10.5 | **<0.001*** | 6.07 | **<0.001*** |
|  | Right pDLPFC | 8.54 | **<0.001*** | 1.11 | 0.984 |
|  | Left FPC | 3.56 | **0.006*** | 3.42 | **0.010*** |
| Sustained | Left Occipital | 8.83 | **<0.001*** | 17.96 | **<0.001*** |
|  | Right Occipital | 5.76 | **<0.001*** | 16.58 | **<0.001*** |
|  | Left anterior Insular | 5.48 | **<0.001*** | -0.97 | 1.000 |
|  | Right anterior Insular | 4.19 | **<0.001*** | -2.14 | 1.000 |
|  | Left IPC | 5.32 | **<0.001*** | 1.98 | 0.507 |
|  | Right IPC | 6.54 | **<0.001*** | 0.25 | 1.000 |
|  | Left Frontal Operculum | 3.14 | 0.0269 | -1.90 | 1.000 |
|  | Right Frontal Operculum | 3.11 | 0.0295 | -2.49 | 1.000 |
|  | SMA /preSMA | 6.48 | **<0.001*** | -1.26 | 1.000 |
|  | Left thalamus | 7.53 | **<0.001*** | 2.63 | 0.122 |
|  | Left Motor | 5.65 | **<0.001*** | 2.24 | 0.314 |
|  | Right Motor | 10.26 | **<0.001*** | 3.29 | **0.016*** |
| Practice + | Left Temporal | 4.24 | **<0.001*** | -2.11 | 1.000 |
|  | Right Temporal | 6.12 | **<0.001*** | -2.74 | 1.000 |
|  | Left Occipital | 10.36 | **<0.001*** | 2.77 | 0.082 |
|  | Right Occipital | 10.36 | **<0.001*** | 8.94 | **<0.001*** |
|  | MOFC | 3.48 | **0.008*** | -3.69 | 1.000 |
| Striatum | Left Anterior Caudate | 6.14 | **<0.001*** | -1.92 | 1.000 |
|  | Right Anterior Caudate | 4.62 | **<0.001*** | -2.78 | 1.000 |
|  | Right Anterior Caudate | 2.43 | 0.206 | -1.91 | 1.000 |

**Supplementary Table 2:** Supplementary analysis of individual ROI within each set for brain activation during rule implementation.

|  | **ROI** | **Practice -** | | **Sustained** | | **Practice+** | |
| --- | --- | --- | --- | --- | --- | --- | --- |
|  |  | **t** | **p** | **t** | **p** | **t** | **p** |
| Practice - | Left LOC | 4.49 | **<0.001*** | 3.57 | **0.006*** | -4.49 | 1.000 |
|  | Right LOC | 3.89 | **0.002*** | 5.98 | **<0.001*** | -3.89 | 1.000 |
|  | Left Parietal | 4.99 | **<0.001*** | 6.81 | **<0.001*** | -4.99 | 1.000 |
|  | Right Parietal | 5.53 | **<0.001*** | 5.58 | **<0.001*** | -5.53 | 1.000 |
|  | Left DLPFC | 4.10 | **0.001*** | 0.8 | 0.999 | -4.1 | 1.000 |
|  | Right DLPFC | 4.82 | **<0.001*** | 1.5 | 0.865 | -4.82 | 1.000 |
|  | Left pDLPFC | 5.45 | **<0.001*** | 3.01 | **0.041*** | -5.45 | 1.000 |
|  | Right pDLPFC | 3.37 | **0.012*** | 1.14 | 0.980 | -3.37 | 1.000 |
|  | Left FPC | 4.04 | **0.001*** | 1.3 | 0.950 | -4.04 | 1.000 |
| Sustained | Left Occipital | -3.42 | 1.000 | 9.57 | **<0.001*** | 3.42 | **0.010*** |
|  | Right Occipital | -4.09 | 1.000 | 6.84 | **<0.001*** | 4.09 | **0.001*** |
|  | Left anterior Insular | 2.41 | 0.212 | 3.75 | **0.003*** | -2.41 | 1.000 |
|  | Right anterior Insular | -0.03 | 1.000 | 3.81 | **0.002*** | 0.03 | 1.000 |
|  | Left IPC | 0.98 | 0.995 | 8.05 | **<0.001*** | -0.98 | 1.000 |
|  | Right IPC | 1.86 | 0.614 | 8.77 | **<0.001*** | -1.86 | 1.000 |
|  | Left Operculum | -0.86 | 1.000 | 9.1 | **<0.001*** | 0.86 | 0.998 |
|  | Right Frontal Operculum | -1.68 | 1.000 | 4.84 | **<0.001*** | 1.68 | 0.755 |
|  | SMA /preSMA | -0.28 | 1.000 | 8.2 | **<0.001*** | 0.28 | 1.000 |
|  | Left thalamus | 2.32 | 0.265 | 4.13 | **0.001*** | -2.32 | 1.000 |
|  | Left Motor | 1.74 | 0.709 | 11.73 | <**0.001*** | -1.74 | 1.000 |
|  | Right Motor | 1.79 | 0.669 | 6.93 | **<0.001*** | -1.79 | 1.000 |
| Practice + | Left Temporal | -0.11 | 1.000 | -2.87 | 1.000 | 0.11 | 1.000 |
|  | Right Temporal | -1.76 | 1.000 | -3.57 | 1.000 | 1.76 | 0.693 |
|  | Left Occipital | -4.06 | 1.000 | -3.81 | 1.000 | 4.06 | **0.001*** |
|  | Right Occipital | -4.32 | 1.000 | -0.02 | 1.000 | 4.32 | **<0.001*** |
|  | MOFC | -2.34 | 1.000 | -3.93 | 1.000 | 2.34 | 0.252 |
| Striatum | Left Anterior Caudate | 0.85 | 0.998 | 0.5 | 1.000 | -0.85 | 1.000 |
|  | Right Anterior Caudate | 1.92 | 0.558 | -0.04 | 1.000 | -1.92 | 1.000 |
|  | Right Anterior Caudate | 0.08 | 1.000 | -0.26 | 1.000 | -0.08 | 1.000 |

**Supplementary Table 3: Peak coordinates of brain activation during attentional reorienting to novel instructions.**

| **Region** | **x** | **y** | **z** | **t** | **p** (FWE corrected) |
| --- | --- | --- | --- | --- | --- |
| Visual Cortex (Medial Occipitotemporal Gyrus) [BA19] | 26 | -54 | -8 | 13.21 | <0.001 |
| Middle Temporal Gyrus Left [BA21] | -52 | -38 | 0 | 7.38 | <0.001 |
| Lateral Parietal Right [BA43] | 60 | -12 | 26 | 5.80 | <0.001 |
| Parietal Operculum Right [BA 48] | 38 | -30 | 18 | 5.64 | 0.001 |
| DLPFC Left [BA9] | -26 | 36 | 36 | 5.04 | 0.009 |
| DLPFC Right [BA9] | 22 | 40 | 40 | 4.79 | 0.024 |
| Parietal Operculum Right [BA48] | 58 | -8 | 14 | 4.74 | 0.031 |
| Insula [BA48] | 30 | -22 | 8 | 4.69 | 0.037 |
| OFC Right [BA11] | 6 | 38 | -12 | 4.68 | 0.038 |
| Frontopolar Cortex Right [BA 10] | 6 | 50 | -8 | 4.67 | 0.040 |
| Parietal Operculum Left  [BA 28] | -38 | -28 | 20 | 4.64 | 0.045 |
| Inferior Parietal Lobules Left [BA48] | -52 | -46 | 32 | 4.63 | 0.047 |
| Premotor Cortex Left [BA6] | -20 | -26 | 56 | 4.62 | 0.047 |
| Putamen Right (extending to Insula) | 28 | -10 | 4 | 4.61 | 0.050 |

**Supplementary Table 4a: Peak coordinates of brain activation during sustained rule encoding.**

| **Region** | **x** | **y** | **z** | **t** | **p** (FWE Corrected) |
| --- | --- | --- | --- | --- | --- |
| Primary & Secondary Visual Cortex [BA17 extends to BA18] | -8 | -90 | 2 | 23.07 | <0.001 |
| DLPFC Left [BA6] | -48 | 8 | 44 | 8.05 | <0.001 |
| Thalamus / Hippocampal | -22 | -28 | -2 | 6.70 | <0.001 |
| Supplementary Motor Area [BA6] | -6 | 8 | 58 | 6.25 | <0.001 |
| Frontal Pole Left [BA45] | -44 | 48 | 4 | 5.87 | <0.001 |
| Thalamus / Hippocampal | 24 | -28 | 2 | 5.70 | <0.001 |
| Posterior DLPFC Right [BA6] | 30 | 2 | 54 | 5.33 | 0.003 |
| Frontal Pole/ Middle Frontal Gyrus  Right [BA45] | 44 | 36 | 32 | 5.09 | 0.007 |

**Supplementary Table 4b:** **Peak coordinates of brain deactivation during sustained rule encoding.**

| **Region** | **x** | **y** | **z** | **t** | **p** (FWE Corrected) |
| --- | --- | --- | --- | --- | --- |
| Middle Temporal Gyrus Left [BA42] | 60 | -42 | 12 | 7.48 | <0.001 |
| Superior Temporal Gyrus Right [BA 22] | -58 | -30 | 12 | 7.45 | <0.001 |
| Corpus Callosum | -20 | -42 | 18 | 7.14 | <0.001 |
| Medial Frontopolar Cortex  [BA10] | -4 | 56 | 18 | 6.43 | <0.001 |

**Supplementary Table 5: Peak coordinates of brain regions that show decreased activity with Practice. Practice -: Contrast across learning stage 1-4: 3,1,-1,-3.**

| **Region** | **x** | **y** | **z** | **t** | **p** (FWE Corrected) |
| --- | --- | --- | --- | --- | --- |
| Parietal Left [BA40] | -48 | -52 | 48 | 6.57 | <0.001 |
| Parietal Right [BA40] | 38 | -54 | 44 | 6.39 | <0.001 |
| Insula Left [BA47] | 34 | 22 | -6 | 6.30 | <0.001 |
| SMA lLeft [BA44] | -34 | 2 | 32 | 6.18 | <0.001 |
| Inferior Occipital Cortex left [BA37] | -46 | -62 | -14 | 6.09 | <0.001 |
| Inferior Frontal Gyrus (operculum) Right [BA44] | 42 | 6 | 28 | 5.56 | 0.001 |
| Inferior Frontal Gyrus (triangularis) Left [BA45] | -44 | 48 | 4 | 5.50 | 0.001 |
| Inferior Occipital Cortex Right [BA37] | 46 | -62 | -16 | 5.15 | 0.006 |
| Middle Frontal Gyrus Right [BA45] | 44 | 38 | 18 | 5.10 | 0.007 |
| Putamen/insula  [BA48] | -28 | 20 | 2 | 4.99 | 0.011 |
| Fusiform Right [BA47] | 32 | -48 | -20 | 4.92 | 0.014 |
| SMA Right [BA8] | 8 | 22 | 50 | 4.90 | 0.016 |
| Middle Frontal Gyrus Right [BA45] | 46 | 32 | 32 | 4.80 | 0.024 |
| SMA Left [BA 32] | -6 | 22 | 46 | 4.63 | 0.047 |

**Supplementary Table 6: Peak coordinates of brain regions that show increase activity with Practice. Practice +: Contrast across learning stage 1-4: -3,-1,1,3.**

| **Region** | **x** | **y** | **z** | **t** | **p** (FWE Corrected) |
| --- | --- | --- | --- | --- | --- |
| Lingual Right [BA18] | 10 | -80 | -2 | 7.22 | <0.001 |
| Superior Occipital Cortex Right [BA19] | -14 | -82 | 28 | 5.82 | <0.001 |
| Cuneus Left [BA18] | 14 | -78 | 26 | 5.25 | 0.004 |
| Calcarine Left [BA17] | -12 | -64 | 12 | 5.07 | 0.008 |
| Medial OFC [BA11] | 0 | 32 | -10 | 5.05 | 0.009 |
| Cuneus Left [BA18] | -4 | -78 | 20 | 4.96 | 0.013 |
| Lingual Left [BA18] | -10 | -66 | 0 | 4.74 | 0.031 |
| Superior Occipital Cortex Right [BA18] | -16 | -94 | 20 | 4.64 | 0.044 |
| Lingual Left [BA18] | -12 | -68 | -6 | 4.61 | 0.050 |

**Supplementary Table 7: Peak coordinates of brain regions that show sustained activity with Practice. Practice Sustained: Contrast across learning stage 1-4: 1,1,1,1.**

| **Region** | **x** | **y** | **z** | **t** | **p** (FWE Corrected) |
| --- | --- | --- | --- | --- | --- |
| SMA Left [BA6] | -4 | -2 | 56 | 14.68 | <0.001 |
| Inferior Parietal Cortex Left [BA2] | -50 | -28 | 46 | 14.12 | <0.001 |
| Lingual Left [BA18] | -8 | -86 | -10 | 12.56 | <0.001 |
| SMA Right [BA6] | 40 | -14 | 54 | 12.07 | <0.001 |
| SMA Left [BA6] | -58 | 6 | 26 | 11.13 | <0.001 |
| SMA Right [BA6] | 60 | 10 | 26 | 7.09 | <0.001 |
| Insula Right [BA48] | 40 | 0 | 12 | 6.09 | <0.001 |
| Insula Left [BA48] | -32 | 14 | 10 | 5.98 | <0.001 |
| Thalamus Left | -10 | -18 | -4 | 5.95 | <0.001 |
| Thalamus Right | 14 | -18 | 4 | 5.14 | 0.006 |

**Supplementary Table 8**

|  | **People with Parkinson’s Unimpaired** | **People with Parkinson’s Impaired** |
| --- | --- | --- |
| n | 11 | 6 |
| Age (years) | 60.6 (5.14) | 62.3 (10.6) |
| Gender (M:F) | 4:7 | 4:2 |
| Disease duration (years) | 5.36 | 4.66 |
| Levodopa equivalent daily dosage (LEDD) | 389 (363) | 556 (372) |
| Hoehn and Yahr stage | 1.75 (0.42) | 2 (0.55) |
| UPDRS (off) | 27.88 (14.90) | 34.33 (7.31) |
| UPDRS III (off) | 33.88 (21.20) | 51 (14.35 |
